# Forgiveness from Emotion Fit: Emotional Frame, Consumer Emotion, and Feeling-Right in Consumer Decision to Forgive

**DOI:** 10.3389/fpsyg.2016.01775

**Published:** 2016-11-15

**Authors:** Yaxuan Ran, Haiying Wei, Qing Li

**Affiliations:** ^1^School of Management, Jinan UniversityGuangzhou, China; ^2^Shenzhen Tourism College, Jinan UniversityShenzhen, China

**Keywords:** emotional frame, consumer emotion, forgiveness, feeling-right, fit

## Abstract

Three studies examine an emotion fit effect in the crisis communication, namely, the interaction between emotional frames of guilt and shame and consumer emotions of anger and fear on consumer forgiveness. Guilt-framing communication results in higher forgiveness than shame-framing for angry consumers, whereas shame-framing communication results in higher forgiveness than guilt-framing for fearful consumers. These effects are driven by consumers’ accessible regulatory foci associated with anger/fear and guilt/shame. Specifically, feelings of anger activate a promotion focus that is represented by guilt frames, while feelings of fear activate a prevention focus that is enacted by shame frames. Compared with emotion non-fit (i.e., anger to shame and fear to guilt), emotion fit (i.e., anger to guilt and fear to shame) facilitates greater feeling-right and consumer forgiveness. The findings offer novel insights for extant literature on emotion, crisis communication, and regulatory focus theory, as well as practical suggestions regarding the emotional frames.

## Introduction

Corporations regularly face a myriad of potential crises, which are low-probability and high-risk events (Yu et al., 2008). Crisis is not a matter of “if” but “when” in corporate life. Therefore, the crisis communication – what and how the company says and does during and after a crisis – is basically essential for garnering consumer forgiveness and restoring consumer-company relations. However, the crisis communication can also make the crisis situation worse (Coombs et al., 2010; Tsarenko and Tojib, 2012). So, how to shape the appropriate strategies in response to corporate crises is critical for firms.

Corporate crises trigger strong and frequent emotions for both consumers and companies (van der Meer and Verhoeven, 2014). In particular, the company is always trying to express “the right tone” during the crisis communication. For example, following the violence and vandalism of the first leg of the Europa League, Feyenoord apologized to Rome and said that “we feel ashamed for the behavior of our citizens in Rome.” In contrast to Feyenoord’s shame-framing response, the Canadian Red Cross “pleaded guilty” in the aftermath of the blood scandal, saying it “is deeply sorry for the injury and death caused to those who were infected by blood or blood products it distributed” in the 1980s and early 1990s. Are these two emotion-framed communications effective in facilitating consumer forgiveness? While considerable crisis communication research has focused on *what* the company should say (Kim et al., 2004; Tsarenko and Tojib, 2015) and *when* the company should respond to the crisis (Frantz and Bennigson, 2005), recently some researchers have begun to pay attention to *how* the company should communicate with consumers, which depicts the way in which emotions the company should frame and express in the crisis communication (Jin, 2010; Kim and Cameron, 2011). Although prior research has shown that communications of negative emotions (e.g., sadness) could substantially have an effect on consumer forgiveness and trust (e.g., ten Brinke and Adams, 2015), previous research has not adequately addressed the impact of distinct negative emotions on consumer forgiveness, nor has it explained those effects. In the current research, we will address this gap and investigate how and why the distinct negative emotions framed in the crisis communication impact consumer forgiveness.

Drawing on prior literature on emotional frames and consumer emotion, we aim to examine how a company’s crisis communication framed in terms of either guilt or shame influences forgiveness for consumers who are experiencing the specific emotions of angry or fear (induced by the crisis or incidental sources). Shame and guilt refer to the self-conscious emotions as they involve perceptions of the self (Tracy and Robins, 2004). These self-conscious emotional frames are frequently used in crisis communication for apologizing, excusing, and pleading, because they often signal that self is wrong (Wolf et al., 2010). Shame and guilt share many similarities, but prior research in consumer psychology has demonstrated the distinct effects of these two emotions on construal level (Han et al., 2014), defensive processing (Agrawal and Duhachek, 2010), and coping processes (Duhachek et al., 2012). In the studies just cited, researchers only examine how consumers experiencing either shame or guilt would react differently, yet little is known about how consumers would perceive and evaluate messages framed in either shame or guilt, especially the emotional frames are not aimed to prime consumers to feel shame or guilt.

In addition, consumer emotions may interact with emotional frames (e.g., shame-framing and guilt-framing) to determine consumer forgiveness. Consumer emotions would affect information processing and decision-making when the onset of emotions is related or incidental to the crisis. Research has suggested that emotional carry-over from the past could cause an implicit cognitive predisposition to appraise subsequent information (Han et al., 2007; Lerner et al., 2004, 2007), such as those apologies framed by the guilt and shame emotions. Still, there is reason to believe that consumer emotions and emotional frames of company may jointly influence consumer forgiveness. Collins’ (2004) interaction ritual chains theory indicates that how one would feel and perceive not only depends on the current situation, but also the previous situations. Based on this rationale, consumer emotion can be seen as a manifestation of the past situation (i.e., the crisis). Specifically, we focus on negative emotions of anger and fear, which are well-established as the most common emotions in crisis (Lerner and Keltner, 2001; Lerner et al., 2003).

In the current research, we show that guilt-framing crisis communication results in higher forgiveness than shame-framing for angry consumers, while shame-framing communication leads to higher forgiveness than guilt-framing for fearful consumers. We refer to the correspondence between consumers’ emotion and companies’ emotional frame as emotion fit. On the consumer’s side, anger, which is induced by a demanding offense against self (Lazarus, 1991), makes people have a blame attribution to the wrongdoer (i.e., the company) and would prefer a promotion-focused coping such as punishment and attack (Nabi, 2003; Carver and Harmon-Jones, 2009). Fear, which is experienced by facing uncertain and existential threats (Lazarus, 1991), makes people more likely to make pessimistic judgment and prefer a prevention-focused coping such as risk-averse orientation, protective solutions, and precautionary plans (Strauman and Higgins, 1987; Lerner and Keltner, 2001; Nabi, 2003). On the company side, guilt-framing is associated with negative behavior evaluation (e.g., “we failed to control quality”) and approach proneness (analogous to promotion focus), whereas shame-framing focuses on negative self-evaluation (e.g., “we failed to keep our promise on quality”) and avoidance proneness (analogous to prevention focus) (Wolf et al., 2010). Thus, drawing on the regulatory focus theory (Higgins, 1997, 1998, 2000; Brockner and Higgins, 2001; Cesario et al., 2004; Santelli et al., 2009), we postulate when the company’s communication frame (i.e., guilt vs. shame) fits with consumers’ emotion (i.e., angry vs. fear) in regulatory focus (i.e., promotion vs. prevention), there would be more perceived feeling-right and in turn greater forgiveness compared with when there is incongruence (i.e., lack of fit).

As a starting point, we review the literature on regulatory focus theory to provide a theoretical basis for the following hypotheses. Examined next is research that, respectively, differentiates angry/fear and guilt/shame in regulatory foci. This is followed by a section that documents how regulatory fit between consumers’ emotion and a company’s emotion in frame increases feeling-right and forgiveness. Finally, we assess evidence that consumers’ perceived feeling-right mediates the main effects.

## Regulatory Focus Theory

Regulatory focus theory delineates how individuals use the basic hedonic principle on approach and avoidance to achieve self-regulatory motivations. At any given point in time, individuals may engage in one of two different types of regulatory focus: promotion focus and prevention focus (Higgins, 1997). Specifically, when promotion focused, one is motivated by ideals, advancement, aspiration, and accomplishment, thereby heightening the salience of attaining gains (i.e., the presence of positive outcomes; Higgins, 1998). When prevention focused, one is then concerned with safety, protection, duties, and responsibility, thereby increasing the salience of avoiding losses (i.e., the presence of negative outcomes; Higgins, 1998).

Although much research treats regulatory foci as stable traits, very smaller research on the antecedents of self-regulation focus shows these regulatory patterns can be made temporarily accessible in situations (e.g., Crowe and Higgins, 1997; Lee et al., 2000). From this perspective, emotions might shape one’s regulatory focus because emotions, both of incidental and message-relevant emotions, are always associated with motivational tendencies and appraisals (Lazarus, 1991; Nabi, 2003). In support of our emotion fit hypotheses, we next document two key premises. First, anger activates a tendency to rely on promotion, and fear induces an inclination focused on prevention. Second, guilt-framing crisis communication delivers a company’s coping strategy associated with a promotion focus and shame-framing indicates a company’s prevention-focused coping strategy.

### Anger/Fear Emotion and Regulatory Focus

Anger and fear, two common negative emotions in risk situations, share many similar consequences. For example, as Lerner and Keltner (2001) suggested, both anger and fear elicit a negative valence appraisal and thus decrease consumers’ purchase intention. However, motivated by the appraisal-tendency framework (Han et al., 2007) that addresses specific emotions can evoke different appraisals, recent research suggests some different effects of the two emotions.

#### Anger Activates a Promotion Focus

Anger arises when “I” or “we” are offended by “the other person, either through neglect or intentionally” (Lazarus, 1991; Li et al., 2014). In a corporate crisis, consumers tend to experience anger when certain company causes an offense against the “self” (myself or ourselves). Literature on physiologic psychology has demonstrated that anger is always associated with an increase in pulse, blood pressure, and increase in epinephrine (Henry, 1986; Cuddy, 2015). In terms of consequential reactions, angry people always process information heuristically (Tiedens and Linton, 2001) and are very likely to blame the company (Han et al., 2007; Carver and Harmon-Jones, 2009). Moreover, feelings of anger drive a desire to take revenge and “fight” (see the customer revenge model; Grégoire et al., 2010; also see Skitka et al., 2006). In short, these physiologic and behavioral reactions suggest that individuals who are experiencing anger tend to pursue promotion-focused goals. From these findings, we theorize that anger can activate a promotion focus.

#### Fear Activates a Prevention Focus

The emotion of fear is induced when one is facing uncertain and existential threat, in which the threat is unpredictable and uncontrollable (Lazarus, 1991; Tiedens and Linton, 2001). In response to a fear-inducing crisis, consumers may benefit from an increase in proinflammatory cytokines that are related with submissive withdrawal (Moons et al., 2010). Additionally, feelings of fear or worried are always associated with uncertain appraisals, which in turn lead people to make pessimistic judgment and precautionary plans (Lerner and Keltner, 2001; Lerner et al., 2003; Skitka et al., 2006). Thus, as Lazarus (1991) proposed, fear would generate a sense of helplessness about protecting the loss. Importantly, Strauman and Higgins (1987) have demonstrated a significant relationship between fear/restless and actual-self/ought-self discrepancy (an index of prevention focus). Together, these findings suggest that experiencing fear leads individuals to adopt a prevention focus.

### Guilt-/Shame-Framing and Regulatory Focus

Guilt and shame emotions in crisis communication could reveal information about the sender (i.e., the company; van der Meer and Verhoeven, 2014), including the sender’s feelings, motives, and concerns. Although much research has demonstrated that both guilt and shame emotions can be elicited by one event and share many similarities (e.g., Tracy and Robins, 2004), recent research has begun to tease apart the different facets of the two emotions (e.g., Dearing et al., 2005; Agrawal and Duhachek, 2010; Duhachek et al., 2012).

#### Guilt Frames Represent Promotion-Focused Coping Strategies

Guilt arises when one - a person or a humanized object, in this case a company - realizes that he or she should take responsibility for the past actions have caused a violation that harms another (Lindsay-Hartz et al., 1995). Guilt frames focus on a specific behavior, such as “we made this mistake,” emphasizing tension, remorse and regret over the “wrongdoing done” (see the review by Tangney et al., 2007). According to research on the behavioral consequences resulted from experiencing guilt (e.g., Wolf et al., 2010; Tangney et al., 2014), the crisis communication framed by guilt emotion could deliver a company’s desire to bring positive changes and show a company’s motivation to repair damage done. Thus, information embedded in the guilt frames indicates a company’s approach tendency that represents a promotion-focused coping strategy.

#### Shame Frames Represent Prevention-Focused Strategies

In contrast, shame results when a person - in this case, a company - judges its wrongdoing as conflicting with its internal standards, norms, and goals, while involving a global negative feeling about the self (Han et al., 2014). Shame frames focus on the deficiency of the company itself (e.g., “we failed to keep our promise on the quality”) with a feeling of diminished, worthless, and powerless (see a review by Tangney et al., 2007). Drawing from previous findings that shame proneness leads individuals to escape, hide, and deny responsibility (e.g., Wolf et al., 2010; Treeby and Bruno, 2012; Tangney et al., 2014), the shame-framing crisis communication would be associated with a tendency to blame other factors for the misconduct and indicate a company’s defensive motivation (Stuewig et al., 2010). As a consequence, shame frames treat as prevention-focused coping strategies because they deliver avoidance messages.

## Emotion Fit, Feeling-Right, and Consumer Forgiveness

Regulatory fit, an important term in regulatory focus theory (Higgins, 1998), occurs when one’s behavior, cognition, or strategic mean naturally is congruent with his or her current regulatory focus (Higgins, 2000; Cesario et al., 2004; Lee and Aaker, 2004; Lee et al., 2010). In the crisis communication context, we postulate that emotion fit results from regulatory fit between the consumer’s emotions and the company’s emotional frames. Previously, we developed the proposition that angry consumers favor promotion-focused strategies that are represented by guilt-framing crisis communications. Therefore, an emotion fit arises between anger and guilt. In the same vein, fearful consumers prefer prevent-focused strategies that are enacted by shame-framing communication, which engenders an emotion fit between fear and shame.

Then, the emotion-fit could result in higher consumer forgiveness. According to McCullough et al. (1998), forgiveness refers to the set of motivational changes whereby one becomes decreasingly motivated to retaliate against an offending partner, decreasingly motivated to maintain estrangement from the offender, and increasingly motivated by conciliation and goodwill toward the offender. That is, when a primary emotion is anger, consumers would prefer a promotion-focused solution such as punishment. The guilt-framing crisis communication focuses on the wrongdoing and shows a company’s promotional motivation to repair damage done. This guilt frames would satisfy angry consumers’ expectations and relieve their emotional tension, hence increasing consumer forgiveness of the company. In contrast, the shame-framing crisis communication that indicates a company’s defensive motivation would be interpreted as the company’s powerless, thereby decreasing consumer forgiveness. When primary emotion is fear, consumers would generate a sense of helplessness and would like to be protected from potential harm. Expressions of shame would communicate a negative feeling about the self. This self-depreciation apology would evoke empathy of fearful consumers, which in turn leads to forgiveness. On the other hand, for fearful consumers, the guilt-framing crisis communication may serve as a cue that the uncertain and existential threat is confirmed and the company is the exact wrongdoer, thereby enhancing feelings of uncertain and then decreasing consumer forgiveness.

When there is an emotion fit, consumers tend to perceive the surroundings around them as more valuable and appropriate and are more likely to trust the company’s communication (Higgins and Silberman, 2001; Laufer and Jung, 2010). Feeling-right is the product of regulatory fit, such that when individuals experience regulatory fit, they feel right about what they are doing. The importance and correctness with which a message is evaluated is conceptualized as feeling of rightness (Cesario et al., 2004), which is subsequently used as evidence in consumer decision to forgive the company (Camacho et al., 2003). Santelli et al. (2009) have suggested that feeling-right would serve as an explanation for why apologies are successful at eliciting forgiveness. Thus, we posit that an emotion fit between a consumer and a company might positively influence the apology’s perceived feeling-right, and then consumer forgiveness, as compared with a mismatch (i.e., lack of fit), which potentially could negatively influence the apology’s perceived feeling-right and consumer forgiveness. Therefore, we hypothesize,

**H1.** For consumers experiencing anger, a guilt-framing crisis communication leads to higher (a) feeling-right and (b) forgiveness than shame-framing.
**H2.** For consumers experiencing fear, a shame-framing crisis communication leads to higher (a) feeling-right and (b) forgiveness than guilt-framing.

As we discussed previously, regulatory foci serve as the mechanism linking consumer emotions and company emotions. Higgins (2000) has argued that regulatory fit increases feeling of rightness because it intensifies and sustains an underlying orientation. That is, the company’s communication strategy that fits consumers’ emotion follows the regulatory focus that is favored by consumers, whereas a non-fit communication strategy follows a different regulatory focus. Furthermore, research has shown that tasks and decisions would be evaluated more positively when they are conducted with regulatory fit (see Higgins, 2000; Cesario et al., 2004). Thus, this line of theorizing predicts that when angry consumers meet a guilt-framing rather than a shame-framing communication, a promotion focus is activated to a greater degree, which in turn results in higher feeling-right and forgiveness. Similarly, when fearful consumers meet a shame-framing rather than a guilt-framing communication, a prevention focus is activated to a greater extent, which in turn leads to higher feeling-right and forgiveness. Therefore, we hypothesize,

**H3.** Angry consumers reading a guilt-framing communication rather than shame-framing engender the activation of promotion focus that, in turn, drives the effects of emotion fit on feeling-right and forgiveness.
**H4.** Fearful consumers reading a shame-framing communication rather than guilt-framing engender the activation of prevention focus that, in turn, drives the effects of emotion fit on feeling-right and forgiveness.

**Table 1** completes the theorizing and predictions. In the three studies that follow, we examine the hypotheses. Study 1 documents the interactive effect of angry/fearful consumer emotion and guilt-/shame-framing communication on feeling-right and forgiveness. Study 2 identifies the underlying process by showing that these effects of consumer emotion and emotional frame on forgiveness are mediated by feeling of rightness and that this feeling-right is the result of enhanced activation of consistent regulatory focus. Finally, in Study 3, we employ a moderation approach to further verify the mediating role of regulatory focus by manipulating promotion focus and prevention focus.

**Table 1:** Hypothesized interaction among consumer emotion, company emotional frame, regulation process, feeling-right, and consumer forgiveness.

Consumer emotion	Company emotion	Regulation process	Information processing	Consumer forgiveness
Anger	Guilt	Fit and promotion focus	High feeling-right	Increased forgiveness
Fear	Guilt	Lack of fit	Low feeling-right	Decreased forgiveness
Anger	Shame	Lack of fit	Low feeling-right	Decreased forgiveness
Fear	Shame	Fit and prevention focus	High feeling-right	Increased forgiveness

## Study 1

The core objective of Study 1 is to test H1 and H2. Study 1 employed a 2 (consumer emotion: anger vs. fear) × 3 (company’s emotional frame: guilt vs. shame vs. no emotion) between-subjects design. Besides measuring self-reported forgiveness, this study also records participants’ time spent on viewing the company’s communication. Viewing time captures the behavioral consequences of feeling-right induced by the company’s crisis communication. According to the basic principles of Implicit Association Tests (IATs; Chang and Mitchell, 2011), viewing time of reading a text is a predictor of perceived incompatibility, which is a manifestation of feeling-right (Camacho et al., 2003). That is, people would read a text faster if they feel the content is “right” and “correct.” Additionally, Chiles and Buslig (2012) have found that perceived incompatibility in apologies could decrease recipients’ forgiveness. Thus, in the context of crisis communication, viewing time is a good estimate of feeling-right for reading the company’s public letter.

### Materials

The stimuli consisted of two piece of fictitious news. The first article described a cell phone explosion issue, aiming to induce either anger or fear. The second article portrayed that the cell phone company responded to the explosion issue with a public letter in either guilt frames or shame frames.

#### Manipulation of Anger/Fear Emotion

According to Nabi (2003), we primed participants to experience anger/fear by emphasizing information related to core relational theme of specific emotion. In the anger condition, the article emphasized the company’s intentional wrongdoing, whereas in the fear condition, the article focused on how other consumers suffer from the company’s wrongdoing.

A pilot study confirmed this emotion priming. Sixty-five graduate students (*M*_age_ = 26.92; 53.8% males) were randomly assigned to either the anger condition or the fear condition. The participants first reported their basic emotions, which include ten items of emotion descriptors (see **Table 2**; Kim and Cameron, 2011). Then the participants read the article and rated a questionnaire. This questionnaire was designed to measure their post-crisis emotions, trustworthiness of the article, and perceived severity, all using 7-point scales ranging from 1 (*not at all*) to 7 (*very much*). See **Table 2** for participants’ emotions before and after reading the article. A series of analysis of variance (ANOVA) revealed that there was a significant difference between angry article condition and fearful article condition for anger emotion (*M*_angry article_ = 2.33 vs. *M*_fearful article_ = 1.31), *F*(1,63) = 6.22, *p* < 0.05, η_p_^2^ = 0.09, and fear emotion (*M*_angry article_ = 1.28 vs. *M*_fearful article_ = 2.35), *F*(1,63) = 5.02, *p* < 0.05, η_p_^2^ = 0.07, but no difference for experiencing sad, disgust, worried, and anxiety (*p*s > 0.18). In addition, no significant differences emerged between the two articles in trustworthiness and severity (*p*s > 0.20).

**Table 2:** Emotions before and after reading the article (Study 1).

Emotions	Before reading *M* (*SD*)	After reading *M* (*SD*)	*M*_diff_	*t-*value	*p*
Angry	1.80 (1.29)	3.72 (1.81)	1.92	9.18	0.00
Disgusted	1.91 (1.45)	3.20 (1.83)	1.29	5.46	0.00
Anxious	2.94 (1.84)	3.62 (1.73)	0.68	3.01	0.00
Fearful	1.87 (1.24)	3.58 (1.73)	1.71	7.12	0.00
Sad	2.31 (1.57)	3.80 (1.87)	1.49	6.18	0.00
Embarrassed	1.86 (1.36)	2.00 (1.30)	0.14	0.71	0.48
Worried	2.83 (1.78)	3.72 (1.80)	0.89	3.15	0.00
Guilty	1.83 (1.33)	1.78 (1.09)	-0.06	-0.35	0.73
Ashamed	1.84 (1.28)	2.02 (1.24)	0.17	0.88	0.38
Contemptuous	1.62 (1.06)	1.62 (1.03)	0.00	0.00	1.00

#### Manipulation of Guilt/Shame Frames

The guilt-framing article was entitled “A GUILTY letter: Apparel Corporate responds to the phone explosion,” while the shame-framing article was entitled “An ASHAMED letter: Apparel Corporate responds to the phone explosion.” We designed the guilt/shame frames based on the research by Duhachek et al. (2012) and another research by van der Meer and Verhoeven (2014). Specifically, in the guilt-framing communication condition, the CEO said “we feel deeply guilty about this serious issue” and “this new phone model failed to be safe” (focusing on the misconduct), and promised to “take all responsibility regarding the event and recall all products” (showing approach-proneness) and finally “express deepest guilt and regret over this issue” again. In the shame-framing communication condition, the CEO said that “we feel deeply ashamed to have allowed this issue to occur” and “we failed in our promise on quality and reliability” (focusing on the trait), and promised that we “will fire the product manager who is in charge of this model and identify other lingering issues in this regard” (showing avoid-proneness) and finally “express our deepest shame, and careful concern for our customers” again. See Appendix A for the details of manipulation.

The manipulation was pretested using fifty-seven participants (*M*_age_ = 21.47; 46% male) who rated the extent to which the company is ashamed of the crisis and the extent to which the company feel guilty of the crisis. Following Zemack-Rugar et al. (2007), guilt consisted of two items (“according to the letter, the company is ashamed/ humiliated,” *r* = 0.77, *p* < 0.001) and shame consisted of three items (“according to the letter, the company is guilt/culpable/remorseful,” α = 0.91), using 7-point scales ranging from 1 (*not at all*) to 7 (*very much so*). Participants exposed to the guilt-framing letter assessed that the company showed more guilt than shame (*M*’s = 5.37 vs. 4.05, respectively), *t*(30) = 4.95, *p* < 0.001, whereas participants exposed to the shame-framing letter assessed that the company expressed shame to a greater extent (*M*’s = 4.79 vs. 3.53, respectively), *t*(25) = 7.72, *p* < 0.001.

### Methods

Two hundred and forty-three undergraduate students (*M*_age_ = 21.26; 51% males) at a public university in China were recruited in exchange for partial course credit and were randomly assigned to the various cells. The study was conducted in a behavioral technology lab on the computer-based interface of E-Prime software. On entering the lab, participants were instructed to sit at individual cubicles with three-foot-high dividers, providing each participant with a private space to complete the experiment independently.

After exposure to one of two crisis articles, participants reported the extent how they feel angry and fearful. Feeling of anger was measured using three items (“I feel angry/irritated/ aggravated,” α = 0.90) and feeling of fear was measured using two items (“I feel fearful/scared,” *r* = 0.42, *p* < 0.01; Dillard and Shen, 2007). Next, participants were randomly assigned to read another article, in which the Apparel Corporate responds to the cell phone explosion issue using one of three frames: guilt-framing, shame-framing, and no emotional framing (control condition). See Appendix A for the details of manipulation. Participants were told to read the company letter until they thoroughly understand it. Once they clicked the start bottom, viewing time was recorded. Upon viewing the letter, participants reported their forgiveness to the Apparel Corporate. Forgiveness was measured by modifying McCullough et al.’s (1998) Transgression-Related Interpersonal Motivation Inventory (TRIM; e.g., “I would be against to the Apparel Corporate”), which is composed of seven items of the Avoidance subscale and five items of the Revenge subscale using scales from 1 (*not at all*) to 7 (*very much so*). We did not incorporate the Benevolence subscale in the TRIM scale as benevolence is a representation of pro-social motivation and always used in the interpersonal context (McCullough et al., 2003). Ample research has demonstrated that the TRIM scale of negative motivations (i.e., Avoidance and Revenge) has good convergent and discriminant validity (McCullough et al., 1998; Santelli et al., 2009). See Appendix B for all measures. Finally, participants provided their demographic information. Upon completion of the experiment, participants were thanked and debriefed.

### Results

#### Manipulation Check

The anger or fear priming was successful; the anger-inducing (vs. fear-inducing) crisis article resulted in significantly more anger (*M*’s = 3.22 vs. 2.17, respectively), *F*(1,237) = 46.87, *p* < 0.001, η_p_^2^ = 0.17, and the fear-inducing (vs. anger-inducing) article resulted in significantly more fear (*M*’s = 3.13 vs. 1.83, respectively), *F*(1,237) = 59.96, *p* < 0.001, η_p_^2^ = 0.20.

#### Consumer Forgiveness

**FIGURE 1:**
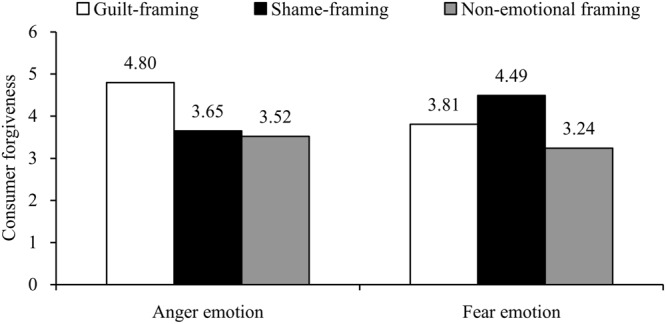
**Interaction effect of consumer emotion and company emotional frame on consumer forgiveness (Study 1)**.

As the TRIM-Avoidance (α = 0.89) and TRIM-Revenge (α = 0.92) subscales showed a high intercorrelation (*r* = 0.80, *p* < 0.001), we created the consumer forgiveness index by computing the average reversed score of TRIM. As expected, a 2 (consumer emotion) × 3 (emotional frame) ANOVA on consumer forgiveness showed a significant interaction effect, *F*(2,237) = 11.20, *p* < 0.001, η_p_^2^ = 0.09 (see **Figure 1**), and a main effect of emotional framing, *F*(2,237) = 12.38, *p* < 0.001, η_p_^2^ = 0.10, but no main effect of consumer emotion, *F* < 1, *p* = 0.36. In terms of the three emotional frame conditions, there was no significant difference between guilt frame and shame frame conditions (*M*_guilt_ = 4.31 vs. *M*_shame_ = 4.07), *F*(1,158) = 1.37, *p* = 0.25, but showed significant differences between emotional frame conditions vs. non-emotional frame condition (*M*_no emotion_ = 3.38), guilt condition vs. no emotion condition, *F*(1,162) = 21.52, *p* < 0.001, η_p_^2^ = 0.12, shame condition vs. no emotion condition, *F*(1,160) = 11.98, *p* < 0.01, η_p_^2^ = 0.07, indicating that participants reading a emotional communication were more likely to forgive the company than those reading a non-emotional communication.

*Post hoc* contrasts revealed that among angry participants, participants who exposed to the guilt-framing communication (*M*_anger-guilt_ = 4.80, *SD* = 1.36) were more likely to forgive the company than those who exposed to the shame-framing communication (*M*_anger-shame_ = 3.65, *SD* = 1.12), *F*(1,237) = 17.11, *p* < 0.001, η_p_^2^ = 0.18, and the no-emotion-framing communication (*M*_anger-no emotion_ = 3.52, *SD* = 1.23), *F*(1,237) = 19.80, *p* < 0.001, η_p_^2^ = 0.20, while there was no difference between guilt-framing and no-emotion-framing condition, *F* < 1, *p* = 0.63. However, among fearful participants, participants who exposed to the shame-framing communication (*M*_fear-shame_ = 4.49, *SD* = 1.34) reported higher forgiveness of the company than those who exposed to the guilt-framing communication (*M*_fear-guilt_ = 3.81, *SD* = 1.11), *F*(1,237) = 6.19, *p* < 0.05, η_p_^2^ = 0.07, whereas participants who exposed to the guilt-framing communication showed greater forgiveness of the company than those who exposed to the no-emotion-framing communication (*M*_fear-no emotion_ = 3.24, *SD* = 1.23), *F*(1,237) = 4.79, *p* < 0.05, η_p_^2^ = 0.06. These findings provided supports for H1b and H2b.

#### Viewing Time

**FIGURE 2:**
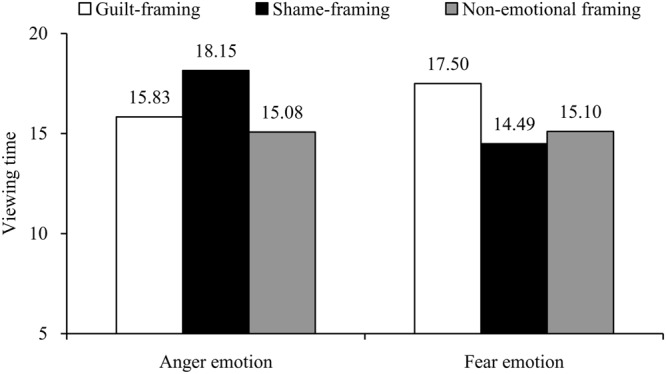
**Interaction effect of consumer emotion and company emotional frame on viewing time (Study 1)**.

Using the viewing time as the dependent variable, another 2 (consumer emotion) × 3 (emotional frame) ANOVA yielded a significant interaction effect, *F*(2,237) = 5.84, *p* < 0.01, η_p_^2^ = 0.05 (see **Figure 2**). Neither consumer emotion nor emotional frame emerged a significant main effect (*p*s > 0.17). Specifically, in the anger condition, participants viewing the shame-framing letter (*M*_anger-shame_ = 18.15 s, *SD* = 5.26) spent more time than those viewing the guilt-framing letter (*M*_anger-guilt_ = 15.83 s, *SD* = 4.46), *F*(1,237) = 4.60, *p* < 0.05, η_p_^2^ = 0.06, and the no-emotion-framing letter as well (*M*_anger-no emotion_ = 15.08 s, *SD* = 3.73), *F*(1,237) = 5.96, *p* < 0.05, η_p_^2^ = 0.07. These results suggest that among angry consumers, shame-framing results in less feeling-right than guilt-framing and no-emotion-framing, thereby supporting H1a. In contrast, in the fear condition, participants viewing the guilt-framing letter (*M*_fear-guilt_ = 17.50 s, *SD* = 6.32) spent less time than those viewing the shame-framing letter (*M*_fear-shame_ = 14.49 s, *SD* = 4.03), *F*(1,237) = 5.12, *p* < 0.05, η_p_^2^ = 0.06, and the non-emotion-framing letter (*M*_fear-no emotion_ = 15.08 s, *SD* = 3.73), *F*(1,237) = 4.54, *p* < 0.05, η_p_^2^ = 0.05. Thus, the results indicate that guilt-framing leads to less feeling-right than shame-framing and no-emotion-framing when consumers are experiencing fear, which thus supports H2a.

### Discussion

The findings of Study 1 support for our proposed theorizing. First, when consumers are experiencing one specific emotion, the emotional communication is more effective than non-emotional communication. Consistent with van der Meer and Verhoeven’s (2014) research, a lack of emotions in company’s response may be interpreted by consumers as a sign, such that an emotionless response implies the absence of organizational involvement and sincerity and may be perceived as cold. Although the viewing time between no emotion condition and the “fit” emotion condition yields no significant difference (e.g., anger-guilt vs. anger-no emotion), the forgiveness between the two conditions is still different.

Second, of central importance, the results demonstrate the interaction between consumer emotion and company emotional frame. Two separate dependent variables together provide convergent evidence in support of the conceptualization; relative to emotion non-fit scenarios (i.e., an angry consumer reads a shame-framing communication and a fearful consumer reads a guilt-framing communication), emotion fit scenarios (i.e., an angry consumer reads a guilt-framing communication and a fearful consumer reads a shame-framing communication) result in higher consumer forgiveness and feeling of rightness.

## Study 2

Study 2 has two goals. The first goal is to examine H3 and H4 that describe the underlying mechanism of emotion fit, so we test whether the regulatory fit, resulted from emotions, facilitates feeling-right and forgiveness. Second, in order to generalize H1 and H2, we examine another situation that anger and fear are unrelated to a crisis. Instead of inducing anger and fear by varying the message frames, we prime participants to recall either angry or fearful events in an ostensibly unrelated experiment before the main valuation of emotional crisis communication.

### Methods

Two hundred undergraduate students (*M*_age_ = 23.11; 56% Males) at a public university in China participated in this experiment in exchange for one course credit. Participants were randomly assigned to conditions in a 2 (consumer emotion: anger vs. fear) × 2 (company’s emotional frame: guilt vs. shame) between-subject design.

The cover story told participants that they would take part in two unrelated studies: the first ostensibly conducted as a psychology experiment and the second conducted as a marketing experiment. At the psychology experiment part, participants were told that this experiment was seeking to understand how people recall. Participants in the anger (fear) condition were requested to recall three past events that made them feel angry (fearful). Specifically, participants were instructed to write down details about one event, in an attempt to recollect how they thought and felt during this episode. This procedure has been shown to be effective for manipulating a specific emotional state (Tiedens and Linton, 2001). After completing the recall task, participants evaluated the extent to which they felt angry (α = 0.94) and fearful (*r* = 0.66, *p* < 0.001), measured in anger and fear scales identical to Study 1.

Participants then proceeded to an ostensibly unrelated marketing experiment. This task followed the same procedure as in Study 1. Participants read a fictitious news article that described the Life water Corporation making a public apology for the sub-standard mineral elements issue. Following the manipulation of Study 1, the shame-framing apology letter was entitled “An ASHAMED LETTER: Life water Corporation responds to spring water issue,” whereas the guilt-framing apology letter was entitled “A GUILTY LETTER: Life water Corporation responds to spring water issue.” Next, participants indicated the extent to which the company is guilty and ashamed of the crisis, their promotion and prevention motivation regarding this event, the feeling-right of the apology content, and their forgiveness of the company. These variables were measured as follow. Guilt (α = 0.90) and shame (*r* = 0.79, *p* < 0.001) were measured as the second pretest in Study 1. By modifying the promotion/prevention scale (Lockwood et al., 2002) to fit the crisis context, promotion and prevention focus was measured using four items, respectively (e.g., promotion focus: “in my point, the current major goal of Life water Corporate should be to take actions to solve the problem,” α = 0.82; prevention focus: “in my point, the current major goal of Life water Corporate should be to avoid the more occurrence of negative issues,” α = 0.80). Feeling-right was measured using two items (e.g., “to what extent do you feel that the Life water’s letter is right,” *r* = 0.45, *p* < 0.01; Cesario et al., 2004). Considering that we only measured the negative motivation of forgiveness in Study 1, we measured forgiveness by adopting a different scale that only consists of four items (e.g., “I would forgive the Life water Corporation,” α = 0.83; Finkel et al., 2002). See the Appendix B for all measures. All items used scales ranging from 1 (*not at all*) to 7 (*very much so*). Finally, participants provided demographic information, following which they were debriefed and thanked.

### Results

#### Manipulation Check

As expected, a 2 (consumer emotion) × 2 (emotional frame) MANOVA on anger and fear showed only a main effect of consumer emotion, such that participants who recalled events that made they feel angry experienced higher anger than those who recalled fearful events (*M*’s = 4.27 vs. 3.39, respectively), *F*(1,196) = 14.30, *p* < 0.001, η_p_^2^ = 0.07, whereas participants who recalled fearful events experienced higher fear than those who recalled angry events (*M*’s = 4.51 vs. 3.74, respectively), *F*(1,196) = 10.59, *p* < 0.01, η_p_^2^ = 0.05.

In addition, another 2 (consumer emotion) × 2 (emotional frame) MANOVA on guilt and shame yielded only a main effect of emotional frame, such that the guilt-framing apology was perceived as more guilty than the shame-framing apology (*M*’s = 4.82 vs. 3.96, respectively), *F*(1,196) = 26.66, *p* < 0.001, η_p_^2^ = 0.12, whereas the shame-framing apology was assessed as more ashamed than the guilt-framing apology (*M*’s = 4.80 vs. 4.20, respectively), *F*(1,196) = 12.03, *p* < 0.01, η_p_^2^ = 0.06. Thus, these results confirmed the manipulations of consumer emotion and emotional frame.

#### Confirmatory Factor Analyses

We conducted confirmatory factor analyses to examine the discriminant validity of the four participant self-reported variables, namely promotion focus, prevention focus, feeling-right and consumer forgiveness. It can be seen from **Table 3** that the Chi-square test of either of the other models shows a significant increase compared to that of the four-factor model, and the four-factor model is obviously better in the other fit indices (Hu and Bentler, 1999). Thus, results showed that the four variables were empirically distinct from each other, representing four distinct constructs.

**Table 3:** Model fit results for confirmatory factor analyses (Study 2).

Variable	χ^2^	*df*	Δ*χ*^2^(Δ*df*)	RMSEA	CFI	TLI	SRMR
(1) Four-factor model	126.39	71	-	0.06	0.95	0.93	0.05
(2) Three-factor model A	427.49	74	301.10(2)	0.16	0.66	0.58	0.15
(3) Three-factor model B	192.95	74	66.56(2)	0.09	0.88	0.86	0.09
(4) Three-factor model C	215.89	74	89.50(2)	0.10	0.86	0.83	0.12
(5) Three-factor model D	149.57	74	23.18(2)	0.07	0.92	0.91	0.06
(6) Two-factor model	511.60	76	385.21(5)	0.17	0.58	0.50	0.19
(7) Single-factor model	612.71	77	486.32(6)	0.19	0.48	0.39	0.16

**N* = 243, All alternative models were compared with the hypothesized four-factor model. All are significant at *p* < 0.001. Three-factor model A combined promotion focus and prevention focus together. Three-factor model B combined promotion focus and feeling-right together. Three-factor model C combined prevention focus and feeling-right together. Three-factor model D combined feeling-right and consumer forgiveness together. Two-factor model combined promotion focus, prevention focus, and feeling-right together. Single-factor model combined promotion focus, prevention focus, feeling-right, and consumer forgiveness together.*

#### Consumer Forgiveness

Central to the emotion fit hypothesis, a 2 (consumer emotion) × 2 (emotional frame) ANOVA on consumer forgiveness showed only a significant interaction effect, *F*(1,196) = 22.75, *p* < 0.001, η_p_^2^ = 0.10. Furthermore, contrast analyses revealed that angry participants indicated greater forgiveness for the company when the apology letter was framed with guilt emotion rather than with shame emotion (*M*_anger-guilt_ = 4.55, *SD* = 1.05 vs. *M*_anger-shame_ = 3.67, *SD* = 1.10), *F*(1,196) = 17.90, *p* < 0.001, η_p_^2^ = 0.15. In contrast, fearful participants indicated greater forgiveness for the company when the apology letter was framed with shame emotion rather than with guilt emotion (*M*_fear-shame_ = 4.31, *SD* = 1.02 vs. *M*_fear-guilt_ = 3.81, *SD* = 0.90), *F*(1,196) = 6.26, *p* < 0.05, η_p_^2^ = 0.06.

#### Promotion and Prevention Foci

We examined the effect of consumer emotion and emotional frame on regulatory foci. As expected, results indicated a main effect of consumer emotion on promotion focus (*M*_anger_ = 4.08, *SD* = 1.21 vs. *M*_fear_ = 3.57, *SD* = 1.05), *F*(1,196) = 10.00, *p* < 0.01, η_p_^2^ = 0.05, and on prevention focus (*M*_anger_ = 3.75, *SD* = 1.02 vs. *M*_fear_ = 4.34, *SD* = 1.12), *F*(1,196) = 15.27, *p* < 0.001, η_p_^2^ = 0.07. There is also a main effect of emotional frame on promotion focus (*M*_guilt_ = 4.16, *SD* = 1.12 vs. *M*_shame_ = 3.50, *SD* = 1.11), *F*(1,196) = 16.60, *p* < 0.001, η_p_^2^ = 0.08, and on prevention focus (*M*_guilt_ = 3.78, *SD* = 1.13 vs. *M*_shame_ = 4.28, *SD* = 1.04), *F*(1,196) = 11.08, *p* < 0.01, η_p_^2^ = 0.05. Therefore, these main effects support the theorizing that anger/fear and guilt/shame are associated with promotion/prevention tendencies.

#### Feeling-Right

We also conducted another 2 (consumer emotion) × 2 (emotional frame) ANOVA on feeling-right. Only the predicted significant two-way interaction emerged, *F*(1,197) = 20.64, *p* < 0.001, η_p_^2^ = 0.10. Further, contrast analyses indicated that participants recalling angry events felt the guilt-framing letter more right than the shame-framing letter (*M*_anger-guilt_ = 4.65, *SD* = 1.19 vs. *M*_anger-shame_ = 3.71, *SD* = 1.21), *F*(1,197) = 16.32, *p* < 0.001, η_p_^2^ = 0.14, whereas those recalling fearful events felt the shame-framing letter more right than the guilt-framing letter (*M*_fear-shame_ = 4.71, *SD* = 1.47 vs. *M*_fear-guilt_ = 4.02, *SD* = 1.20), *F*(1,197) = 6.23, *p* < 0.05, η_p_^2^ = 0.06.

#### Sequential Mediation Analyses

As H3a and H3b represent a case of moderated mediation, we analyzed the mediating role of promotion and prevention focus, respectively (cf. Muller et al., 2005). We expected that in each emotion condition (promotion focus for anger vs. prevention focus for fear), the promotion/prevention focus mediated the relationship between emotional frame and feeling-right, which subsequently influence consumer forgiveness.

**FIGURE 3:**
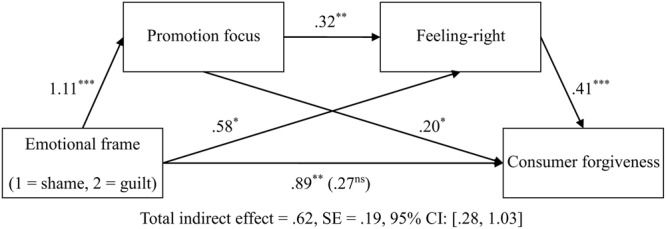
**The sequential mediating model in the anger condition (Study 2).** ^ns^*p* > 0.1, ^∗^*p* < 0.05, ^∗∗^*p* < 0.01, ^∗∗∗^*p* < 0.001.

First, focusing on the anger condition, the bias-corrected bootstrap analyses (Model 6, 1000 resamples; Hayes, 2013) provided support for the sequential mediation chain: emotional frame → promotion focus → feeling-right → consumer forgiveness, indirect effect path = 0.15, *SE* = 0.08, 95% CI: [0.0407, 0.3729] (see **Figure 3** for complete path coefficients and total indirect effect), but not for the prevention focus pathway: emotional frame → prevention focus → feeling-right → consumer forgiveness, indirect effect path = 0.00, *SE* = 0.03, 95% CI: [-0.0488, 0.0965], thereby supporting H3.

**FIGURE 4:**
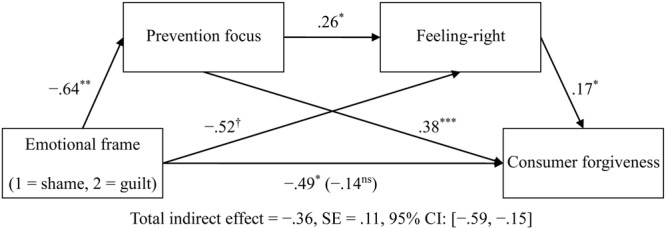
**The sequential mediating model in the fear condition (Study 2).** ^ns^*p* > 0.1, ^†^*p* < 0.1, ^∗^*p* < 0.05, ^∗∗^*p* < 0.01, ^∗∗∗^*p* < 0.001.

Next, we focused on the fear condition and examined the indirect effect of emotional frame upon feeling-right and then consumer forgiveness through prevention focus, not through promotion focus. Of central importance, the bias-corrected bootstrap analyses (Model 6, 1000 resamples) supported the sequential mediation chain through the prevention focus pathway: emotional frame → prevention focus → feeling-right → consumer forgiveness, indirect effect path = -0.03, *SE* = 0.02, 95% CI: [-0.1008, -0.0047] (see **Figure 4** for complete path coefficients and total indirect effect), but did not support the mediation chain through the promotion focus pathway, indirect effect path = 0.01, *SE* = 0.01, 95% CI: [-0.0144, 0.0534], which thus supports H4.

### Discussion

The results in Study 2 support our conceptualization. First, consistent with our basic theorizing, the results provide evidence in support of the key linkages between anger/fear and promotion/prevention activation and between guilt/shame and promotion-/prevention-focused strategies. Second, the results replicate the patterns of emotion fit effect found in Study 1. Third, the results support the sequential mediation, such that the emotional framings interact with consumer emotions to influence consumer’s regulatory focus and hence feeling-right and forgiveness.

## Study 3

The objective of Study 3 is to provide further process evidence of promotion and prevention focus. By manipulating promotion and prevention focus directly, we strive to test the role of regulatory focus in the emotion fit effects using a moderation-of-process design to complement the mediation approach used in Study 2 (cf., Spencer et al., 2005). When a specific regulatory focus is made accessible, we should find that the fit between focus and emotional frame predicts forgiveness, which is consistent with the findings of Santelli et al. (2009). More specifically, the guilt-framing communication should result in greater forgiveness and feeling-right than the shame-framing communication for promotion-focused consumers, while the shame-framing communication should result in greater forgiveness and feeling-right than the guilt-framing communication for prevention-focused consumers.

### Methods

Two hundred and ninety-five undergraduate students (*M*_age_ = 21.67; 46% Males) at a public university in China participated in this experiment in exchange for ¥15 (approximately 2.32 US dollars). Participants were randomly assigned to conditions in a 2 (consumer emotion: anger vs. fear) × 2 (regulatory focus: promotion vs. prevention) × 2 (company’s emotional frame: guilt vs. shame) between-subject design.

As a cover story, participants were told that they would take part in three unrelated studies: the first would be a psychology experiment, the second would be a survey held by the Student Union, and the third would be a news evaluation held by the marketing department. The first task followed the same procedure as the first one in Study 2, involving recalling an experience of either anger or fear. Then, participants indicated how they feel angry (α = 0.93) and fearful (*r* = 0.41, *p* < 0.01).

Proceeding to the second study, participants were told that the Student Union was collecting students’ status quo. On this pretense, participants in the promotion-priming condition were asked to describe their current hopes and aspirations and why aspirations are important to people, whereas participants in the prevention-priming condition were asked to describe their current sense of duty and obligation and why obligations are important to people (cf., Higgins, 1998; Higgins and Silberman, 2001).

Next, participants moved to the news evaluation task, which is designed in a manner identical to Study 2. Upon reading the news, participants indicated their feeling-right about the apology (*r* = 0.62, *p* < 0.01) and their forgiveness of the company (α = 0.89), assessed in a manner identical to Study 2. Finally, participants provided demographic information and were thanked and debriefed.

### Results

#### Manipulation Check

Participants in the anger condition experienced greater anger than those in the fear condition (*M*’s = 4.46 vs. 3.77, respectively), *F*(1,293) = 11.87, *p* < 0.01, whereas participants in the fear condition experienced greater fear than those in the anger condition (*M*’s = 4.40 vs. 3.59, respectively), *F*(1,293) = 24.45, *p* < 0.001, thus confirming that the emotion manipulation was successful.

#### Consumer Forgiveness

We performed a 2 (consumer emotion) × 2 (regulatory focus) × 2 (emotional frame) ANOVA on consumer forgiveness. Results only revealed a two-way interaction effect between regulatory focus and emotional frame, *F*(1,287) = 16.19, *p* < 0.001, η_p_^2^ = 0.05. Neither the other interactions nor three main effects were significant (*p*s > 0.08).

**FIGURE 5:**
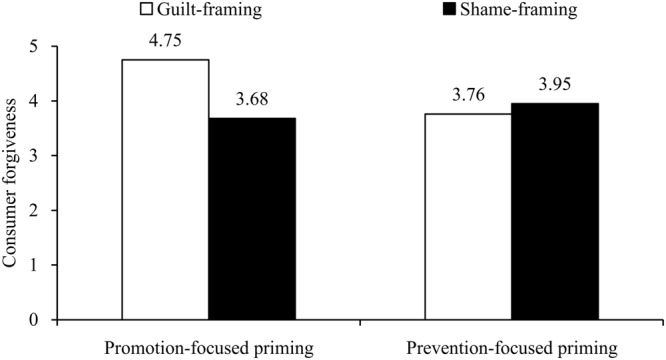
**Interaction effect of emotion and regulatory focus on forgiveness in the anger condition (Study 3)**.

**FIGURE 6:**
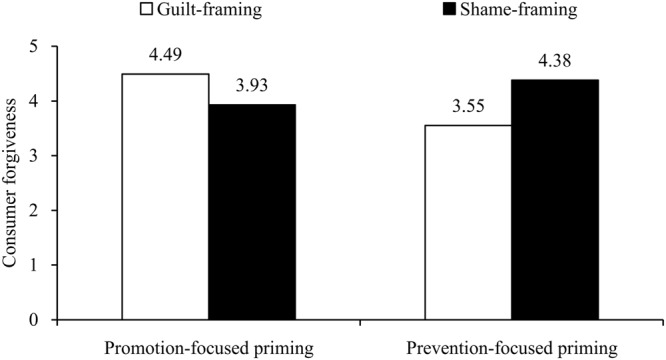
**Interaction effect of emotion and regulatory focus on forgiveness in the fear condition (Study 3)**.

*Post hoc* contrasts indicated that within the promotion-priming condition, participants reported higher forgiveness for the company using guilt frames rather than shame frames (*M*_promotion-guilt_ = 4.62, *SD* = 1.38 vs. *M*_promotion-shame_ = 3.80, *SD* = 1.40), *F*(1,287) = 13.07, *p* < 0.001, η_p_^2^ = 0.08. Conversely, within the prevention-priming condition, participants reported higher forgiveness for the company using shame frames rather than guilt frames (*M*_prevention-shame_ = 4.16, *SD* = 1.49 vs. *M*_prevention-guilt_ = 3.66, *SD* = 1.40), *F*(1,287) = 4.33, *p* < 0.05, η_p_^2^ = 0.03. Further, the significant interaction between regulatory focus and emotional frame still emerged in the anger, *F*(1,145) = 8.16, *p* < 0.01, η_p_^2^ = 0.05 (see **Figure 5**), and fear conditions, respectively, *F*(1,142) = 8.05, *p* < 0.01, η_p_^2^ = 0.05 (see **Figure 6**).

#### Feeling-Right

Consistent with the patterns of consumer forgiveness, another 2 (regulatory focus) × 2 (consumer emotion) × 2 (emotional frame) ANOVA on feeling-right showed a significant two-way interaction effect between regulatory focus and emotional frame, *F*(1,287) = 27.42, *p* < 0.001, η_p_^2^ = 0.09. Regardless of the anger and fear condition, there was always a significant interaction between regulatory focus and emotional frame in the anger condition, *F*(1,145) = 14.31, *p* < 0.001, η_p_^2^ = 0.09, and in the fear condition, *F*(1,142) = 13.14, *p* < 0.001, η_p_^2^ = 0.09. Further, no significant other interactions or three main effects emerged (*p*s > 0.09).

### Discussion

The findings of Study 3 show that activation of regulatory focus drives the effects of emotion fit on forgiveness. When a specific regulatory focus is activated, regardless of consumer emotion, the fit between regulatory focus and emotional frame predicts feeling-right and forgiveness. That is, the promotion focus drives the effects of guilt frame on forgiveness and feeling-right, while the prevention focus underlies the effects of shame frame on forgiveness and feeling-right. Together with the findings of Study 2, these results converge in support of the regulatory focus mechanism in emotion fit.

## General Discussion

The current research suggests that in the corporate crises emotional frame of company interacts with consumer emotion to determine consumer forgiveness. In general, this research combines three different research streams - anger/fear, guilt/shame, and regulatory focus - into a comprehensive framework regarding how and why emotional frame impacts consumer forgiveness. Foremost, the effect of emotional frame (guilt-framing vs. shame–framing) on consumer forgiveness depends on consumer emotion — whether consumers feel angry or fearful. Across three experiments, we show that for consumers who are experiencing anger, the guilt-framing communication could result in greater forgiveness than shame-framing communication, whereas for consumers who are experiencing fear, the shame-framing communication could result in greater forgiveness than guilt-framing communication (Studies 1 and 2). In addition, the emotion fit effects also exist when consumer emotion is not related with the crisis (Studies 2 and 3). Further, we pinpoint the specific mechanisms that underlie these effects. Specifically, drawing on the regulatory focus theory, we show that consumers feeling anger (fear) prefer promotion-focused (prevent-focused) strategies that are enacted by guilt-framing (shame-framing) communication, thereby enhancing their feeling-right and then their forgiveness (Studies 2 and 3).

### Theoretical Contributions

First, this research contributes to the literature on crisis communication by identifying a new driver of communication effectiveness, namely, guilt/shame emotional frames. Prior empirical investigations of crisis communication have mainly focused on *what* the company says in the communication (Kim et al., 2004; Tsarenko and Tojib, 2015) and *when* the company responds to the crisis (Frantz and Bennigson, 2005). Motivated by the idea that “attitude is everything,” scholars have increasingly recognized the important role of emotion in crisis communication (Li et al., 2014). Claeys and Cauberghe (2014) found that crisis response with an emotional appeal influence consumers’ interpretation of crisis, which may subsequently have an effect on consumer forgiveness. In addition, Kim and Cameron (2011) also showed that the presence (vs. absence) of a suitable emotional communication increases consumer trust. This series of studies has demonstrated the importance of emotional appeals, leaving the question regarding which specific emotion the company should frame and express in the crisis communication not fully explored. A recent study by ten Brinke and Adams (2015) indicated that corporate apologies with a negative emotion (i.e., sadness) would positively impact perceived sincerity as apposed to apologies with a positive emotion (i.e., happiness). However, this valence-based approach cannot account for the distinct effects of emotions similar in valence (Han et al., 2007). Both guilt and shame are negative and self-conscious emotions; nevertheless, due to their distinct behavioral implications (Dearing et al., 2005; Agrawal and Duhachek, 2010; Duhachek et al., 2012; Tangney et al., 2014), we expected and identified the distinct effects of guilt-framing and shame-framing on consumer forgiveness. To the best of our knowledge, this is the first research to examine the relative effectiveness of guilt-framing and shame-framing on consumer forgiveness.

Next, these findings also add to a burgeoning body of research on guilt and shame by providing the underlying mechanism of promotion and prevention foci. Previous research has shown that the differential effects of guilt and shame on construal level (Han et al., 2014), defensive processing (Agrawal and Duhachek, 2010), coping processing (Duhachek et al., 2012), and appraisal tendencies (Han et al., 2014) for people who are objects of emotions. Yet the notion how guilt and shame frames impact consumers has received little attention. By demonstrating the interaction effect of emotional frame and consumer emotion on forgiveness is driven by promotion/prevention focus and feeling-right, we provide a comprehensive picture in understanding effects of shame/guilt frame on consumer forgiveness. Although Duhachek et al. (2012) have found that problem-focused/emotion-focused coping drives the interactive effect of shame/guilt and gain/loss on message persuasiveness, we provide a distinct explanation of promotion/prevention focus. That is, problem-focused coping focuses on a problem-solving approach (e.g., rational thinking and action) and emotional-focused coping focuses on an emotional approach to reduce stress (e.g., emotional venting), whereas both promotion and prevention foci aim at solving problems but use different ways.

Furthermore, at a broader level, our work contributes to research on emotion by providing an early inquiry into the emotion fit effects. We show that an emotion fit arises between consumers’ emotion and companies’ emotion, such that consumers’ anger fits the company’s guilt and consumers’ fear fits the company’s shame. Indeed, previous research has documented some phenomenon related with emotion match. For example, Ludwig et al. (2013) proposed the linguistic style match, such that a communicator who is using emotional linguistic style would more prefer another communicator who is using emotional (vs. rational) linguistic style. Moreover, Bower (1981) suggested the mood congruity effect that people always look for mood-congruent others but avoid mood-incongruent others (also see Lee et al., 2013). Whereas these aforementioned studies have only focused on whether the two communicators have the same emotions (e.g., a sad person to another sad person), we investigate the fit between two different specific emotions (i.e., anger to guilt and fear to shame). Thus, the findings contribute to the emotion research and open the door to subsequent investigations of more emotion fit phenomenon.

Lastly, the current study extends regulatory focus theory to the emotion domain. Literature on regulatory focus theory mainly focuses on the cognition domain, such as consumer goals (Lee et al., 2010), gain/loss frames (Lee and Aaker, 2004), and self-construal (Lee et al., 2000). Although Brockner and Higgins (2001) have suggested that regulatory foci can influence emotions, they did not imply that emotions can predict regulatory foci. Our results show that the basic principles of regulatory focus theory (e.g., regulatory fit), which are well established in the domain of motivation and cognition, also hold for emotions.

### Practical Implications

Guilt and shame emotions are frequently used in the crisis communication as a consequence of begging forgiveness. Our research provides insight into how company’s emotional messages and consumers’ emotions jointly influence the effectiveness of crisis communication. When a crisis occurs, the company should think carefully about the emotional frames used in the public letter and consider clearly consumer emotions. Two recommendations follow. First, if a crisis elicits a strong emotional response from consumers, managers should use emotional frames and tactics rather than more “rational” approaches (cf. Luo and Yu, 2015). Second, managers should try to pin down the emotions of majority consumers, and then use the specific “fit” emotional frame to respond to the crisis. For instance, when anger is the dominant reaction toward a crisis, using an apology letter full of feeling guilty is effective to garner consumers’ forgiveness. Yet, in managing a crisis that primarily evokes fear, providing an apology letter full of feeling ashamed of the relevant actions might be more important. Although a crisis undoubtedly triggers numerous emotions, the primary emotion could be predicted as emotions are related with the nature of the crisis. According to Jin’s (2010) integrated crisis mapping model, more anger would be experienced as a function of perceived high crisis predictability and high crisis controllability, and more fear would be experienced as a function of perceived low crisis predictability and low crisis controllability.

An additional practical implication of our research lies in the findings that specific regulatory focus drives feeling-right and forgiveness. On the basis of using emotional frames in the crisis communication, managers could further increase consumers’ forgiveness of the company by highlighting information regarding a particular regulatory focus. For example, the shame-framing apology letter can include a statement such as “protecting you against loss is our primary obligation,” which is an example of prevention focus; the guilt-framing apology letter can include a statement such as “we strive to make world-class products that deliver the best experience possible to our customers,” which represents a promotion focus. This statement could make the proper regulatory focus salient, thereby facilitating consumers’ acceptance of the apology and consumers’ forgiveness. All in all, managers need to understand the regulatory focus activated by consumer emotions and design public letters that aid the specific regulatory focus to maximize the effectiveness of crisis communication.

### Limitations and Future Research

The present research has several limitations that suggest a number of potentially future research opportunities. Across the three experiments, we manipulated emotional frames using news articles. In reality, however, the misbehaving company also makes apologies using conference press. Considering that a lot of visual characteristics could influence consumer judgment (e.g., colors, subtle expressions), we only employ the content frames manipulation to examine the hypotheses. Also, our research only involves student participants and all participants were only Chinese nationals, which may raise external validity. Future research can further examine the emotion fit effect across different cultures and enhanced generalizability. Next, three experiments only examined the self-report forgiveness, which cannot directly predict consumers’ real behavior. However, our findings were replicated using two different scales to measure forgiveness, suggesting the robustness of effects. Finally, inputs into forgiveness other than the emotional frames might exert similar effects, such as those that arise in the content of communication, the company-consumer relationship, the intentionality of crisis, the type of crisis (e.g., performance-related crisis vs. values-related crisis), and the severity of crisis. These salient predictors of forgiveness are likely to present important boundary conditions for our results and their effects.

Another avenue for future research is to explore other emotion fit effects. We only found the fit between anger and guilt and between fear and shame, yet there are many other emotions. For example, how does a consumer who is experiencing pride react to a happy vs. a sad song? Given anxiety and anger are the two common emotions embedded in the negative product reviews (Yin et al., 2014), how does an anxious (vs. an angry) review influence a sad consumer’s perceived helplessness? And for two donation advertisements that one highlights hope and another delivers love, which one is more effective for a consumer who feels embarrassed? These interesting issues merit further examination.

The issue of mediators was also addressed in the current research. Although Studies 2 and 3 have demonstrated the mediating role of promotion/prevention focus, other factors could have been influenced in the casual chain predicting forgiveness as well. That is, does emotion also impact how consumers feel about other variables that are typically involved in the forgiving process? For instance, anger and fear may influence consumers’ attributions on crisis (Han et al., 2007; Kim and Cameron, 2011), thereby impacting how consumers evaluate the emotional frames. The current research only shed light upon one regulatory focus mechanism, so further research could consider other underlying mechanisms and examine to what extent these different mechanisms can account for the emotion fit effects.

## Conclusion

This paper has studied emotion fit when companies must conduct crisis communication. More specifically, the interaction between emotional frames of guilt and shame with consumer emotions of anger and fear and the effect on consumer forgiveness are examined. Guilt-framing communication results in higher forgiveness than shame-framing for angry consumers, whereas shame-framing communication results in higher forgiveness than guilt-framing for fearful consumers. Compared with emotion non-fit (i.e., anger to shame and fear to guilt), emotion fit (i.e., anger to guilt and fear to shame) facilitates greater feeling-right and consumer forgiveness. This means that managers should try to pin down the emotions of majority consumers, and then use the specific “fit” emotional frame to respond to the crisis. The findings offer novel insights for extant literature on crisis communication, emotion, and regulatory focus theory. It further underscores the importance in understanding different types of emotion and the responses they can elicit (Li et al., 2014) as well as providing practical suggestions regarding the emotional frames.

## Author Contributions

YR and HW designed the experiments. YR and QL conducted the experiment and analyzed the data. YR wrote the manuscript. HW and QL edited the manuscript.

## Conflict of Interest Statement

The authors declare that the research was conducted in the absence of any commercial or financial relationships that could be construed as a potential conflict of interest.

## References

[B1] Agrawal N. Duhachek A. (2010). Emotional compatibility and the effectiveness of antidrinking messages: a defensive processing perspective on shame and guilt. *J. Mark. Res.* 47 263–273. 10.1509/jmkr.47.2.263

[B2] Bower G. H. (1981). Mood and memory. *Am. Psychol.* 36 129–148. 10.1037/0003-066X.36.2.129 7224324

[B3] Brockner J. Higgins E. T. (2001). Regulatory focus theory: implications for the study of emotions at work. *Organ. Behav. Hum. Decis. Process.* 86 35–66. 10.1006/obhd.2001.2972

[B4] Camacho C. J. Higgins E. T. Luger L. (2003). Moral value transfer from regulatory fit: what feels right is right and what feels wrong is wrong. *J. Pers. Soc. Psychol.* 84 498–510. 10.1037/0022-3514.84.3.498 12635912

[B5] Carver C. S. Harmon-Jones E. (2009). Anger is an approach-related affect: evidence and implications. *Psychol. Bull.* 135 183–204. 10.1037/a0013965 19254075

[B6] Cesario J. Grant H. Higgins E. T. (2004). Regulatory fit and persuasion: transfer from “feeling right”. *J. Pers. Soc. Psychol.* 86 388–404. 10.1037/0022-3514.86.3.388 15008644

[B7] Chang B. P. Mitchell C. J. (2011). Discriminating between the effects of valence and salience in the implicit association test. *Q. J. Exp. Psychol.* 64 2251–2275. 10.1080/17470218.2011.586782 21722058

[B8] Chiles B. W. Buslig A. L. (2012). “I’m, uhh, Sorry”: the influence of fluency and communication competence on perceptions of apologies. *Commun. Theater Assoc. Minn. J.* 39 66–85.

[B9] Claeys A. S. Cauberghe V. (2014). What makes crisis response strategies work? The impact of crisis involvement and message framing. *J. Bus. Res.* 67 182–189. 10.1016/j.jbusres.2012.10.005

[B10] Collins R. (2004). *Interaction Ritual Chains.* Princeton, NJ: Princeton University Press.

[B11] Coombs W. T. Frandsen F. Holladay S. J. Johansen W. (2010). Why a concern for apologia and crisis communication? *Corp. Commun.* 15 337–349. 10.1108/13563281011085466

[B12] Crowe E. Higgins E. T. (1997). Regulatory focus and strategic inclinations: promotion and prevention in decision-making. *Organ. Behav. Hum. Decis. Process.* 69 117–132. 10.1006/obhd.1996.2675

[B13] Cuddy A. (2015). *Presence: Bringing Your Boldest Self to Your Biggest Challenges.* New York, NY: Little, Brown and Company.

[B14] Dearing R. L. Stuewig J. Tangney J. P. (2005). On the importance of distinguishing shame from guilt: relations to problematic alcohol and drug use. *Addict. Behav.* 30 1392–1404. 10.1016/j.addbeh.2005.02.002 16022935 PMC3106346

[B15] Dillard J. P. Shen L. (2007). “Self-report measures of discrete emotions,” in *Handbook of Research on Electronic Surveys and Measurements* eds Reynolds R. A. Woods R. Baker J. D. (Hershey, PA: Ideal Group Publishing) 330–333. 10.4018/978-1-59140-792-8.ch044

[B16] Duhachek A. Agrawal N. Han D. (2012). Guilt versus shame: coping, fluency, and framing in the effectiveness of responsible drinking messages. *J. Mark. Res.* 49 928–941. 10.1509/jmr.10.0244

[B17] Finkel E. J. Rusbult C. E. Kumashiro M. Hannon P. A. (2002). Dealing with betrayal in close relationships: does commitment promote forgiveness? *J. Pers. Soc. Psychol.* 82 956–974. 10.1037/0022-3514.82.6.956 12051583

[B18] Frantz C. M. Bennigson C. (2005). Better late than early: the influence of timing on apology effectiveness. *J. Exp. Soc. Psychol.* 41 201–207. 10.1016/j.jesp.2004.07.007

[B19] Grégoire Y. Laufer D. Tripp T. M. (2010). A comprehensive model of customer direct and indirect revenge: understanding the effects of perceived greed and customer power. *J. Acad. Mark. Sci.* 38 738–758. 10.1007/s11747-009-0186-5

[B20] Han D. Duhachek A. Agrawal N. (2014). Emotions shape decisions through construal level: the case of guilt and shame. *J. Consum. Res.* 41 1047–1064. 10.1086/678300

[B21] Han S. Lerner J. S. Keltner D. (2007). Feelings and consumer decision making: the appraisal-tendency framework. *J. Consum. Psychol.* 17 158–168. 10.1016/S1057-7408(07)70023-2

[B22] Hayes A. F. (2013). *Introduction to Mediation, Moderation, and Conditional Process Analysis.* New York, NY: Guilford.

[B23] Henry J. P. (1986). “Neuroendocrine patterns of emotional response,” in *Emotion: Theory, Research and Experience* Vol. 3 eds Plutchik R. Kellerman H. (Orlando, FI: Academic Press) 37–60. 10.1016/B978-0-12-558703-7.50008-5

[B24] Higgins E. T. (1997). Beyond pleasure and pain. *Am. Psychol.* 52 1280–1300. 10.1037/0003-066X.52.12.1280 9414606

[B25] Higgins E. T. (1998). Promotion and prevention: regulatory focus as a motivational principle. *Adv. Exp. Soc. Psychol.* 30 1–46. 10.1016/S0065-2601(08)60381-0

[B26] Higgins E. T. (2000). Making a good decision: value from fit. *Am. Psychol.* 55 1217–1230. 10.1037//0003-066X.55.11.1217 11280936

[B27] Higgins E. T. Silberman I. (2001). “Development of regulatory focus: promotion and prevention as ways of living,” in *Motivation and Self-regulation Across the Lifespan* eds Heckhausen J. Dweck C. S. (New York, NY: Cambridge University Press) 10.1017/CBO9780511527869

[B28] Hu L. T. Bentler P. M. (1999). Cutoff criteria for fit indexes in covariance structure analysis: conventional criteria versus new alternatives. *Struct. Equ. Modeling* 6 1–55. 10.1080/10705519909540118

[B29] Jin Y. (2010). Making sense sensibly in crisis communication: how publics’ crisis appraisals influence their negative emotions, coping strategy preferences, and crisis response acceptance. *Commun. Res.* 37 522–552. 10.1177/0093650210368256

[B30] Kim H. J. Cameron G. T. (2011). Emotions matter in crisis: the role of anger and sadness in the publics’ response to crisis news framing and corporate crisis response. *Commun. Res.* 38 826–855. 10.1108/13563281011085529

[B31] Kim P. H. Ferrin D. L. Cooper C. D. Dirks K. T. (2004). Removing the shadow of suspicion: the effects of apology versus denial for repairing competence- versus integrity-based trust violations. *J. Appl. Psychol.* 89 104–118. 10.1037/0021-9010.89.1.104 14769123

[B32] Laufer D. Jung J. M. (2010). Incorporating regulatory focus theory in product recall communications to increase compliance with a product recall. *Public Relat. Rev.* 36 147–151. 10.1016/j.pubrev.2010.03.004

[B33] Lazarus R. S. (1991). *Emotion and Adaptation.* New York, NY: Oxford University Press.

[B34] Lee A. Y. Aaker J. L. (2004). Bringing the frame into focus: the influence of regulatory fit on processing fluency and persuasion. *J. Pers. Soc. Psychol.* 86 205–218. 10.1037/0022-3514.86.2.205 14769079

[B35] Lee A. Y. Aaker J. L. Gardner W. L. (2000). The pleasures and pains of distinct self-construals: the role of interdependence in regulatory focus. *J. Pers. Soc. Psychol.* 78 1122–1134. 10.1037/0022-3514.78.6.1122 10870913

[B36] Lee A. Y. Keller P. A. Sternthal B. (2010). Value from regulatory construal fit: the persuasive impact of fit between consumer goals and message concreteness. *J. Consum. Res.* 36 735–747. 10.1086/605591

[B37] Lee C. J. Andrade E. B. Palmer S. E. (2013). Interpersonal relationships and preferences for mood-congruency in aesthetic experiences. *J. Consum. Res.* 40 382–391. 10.1086/670609

[B38] Lerner J. S. Gonzalez R. M. Small D. A. Fischhoff B. (2003). Effects of fear and anger on perceived risks of terrorism a national field experiment. *Psychol. Sci.* 14 144–150. 10.1111/1467-9280.01433 12661676

[B39] Lerner J. S. Han S. Keltner D. (2007). Feelings and consumer decision making: extending the appraisal-tendency framework. *J. Consum. Psychol.* 17 181–187. 10.1016/S1057-7408(07)70027-X

[B40] Lerner J. S. Keltner D. (2001). Fear, anger, and risk. *J. Pers. Soc. Psychol.* 81 146–159. 10.1037/0022-3514.81.1.146 11474720

[B41] Lerner J. S. Small D. A. Loewenstein G. (2004). Heart strings and purse strings: carryover effects of emotions on economic decisions. *Psychol. Sci.* 15 337–341. 10.1111/j.0956-7976.2004.00679.x 15102144

[B42] Li Y. Ashkanasy N. M. Ahlstrom D. (2014). The rationality of emotions: a hybrid process model of decision-making under uncertainty. *Asia Pac. J. Manag.* 31 293–308. 10.1007/s10490-012-9341-5

[B43] Lindsay-Hartz J. DeRivera J. H. Mascolo M. F. (1995). “Differentiating guilt and shame and their effects on motivation,” in *Self-Conscious Emotions: The Psychology of Shame, Guilt, Embarrassment, and Pride* eds Tangney J. P. Fischer K. W. (New York, NY: Guilford Press) 274–300.

[B44] Lockwood P. Jordan C. H. Kunda Z. (2002). Motivation by positive or negative role models: regulatory focus determines who will best inspire us. *J. Pers. Soc. Psychol.* 83 854–864. 10.1037/0022-3514.83.4.854 12374440

[B45] Ludwig S. De Ruyter K. Friedman M. Brüggen E. C. Wetzels M. Pfann G. (2013). More than words: the influence of affective content and linguistic style matches in online reviews on conversion rates. *J. Mark.* 77 87–103. 10.1509/jm.11.0560

[B46] Luo J. Yu R. (2015). Follow the heart or the head? The interactive influence model of emotion and cognition. *Front. Psychol.* 6:573 10.3389/fpsyg.2015.00573 PMC4422030 25999889

[B47] McCullough M. E. Fincham F. D. Tsang J. A. (2003). Forgiveness, forbearance, and time: the temporal unfolding of transgression-related interpersonal motivations. *J. Pers. Soc. Psychol.* 84 540–557. 10.1037//0022-3514.84.3.540 12635915

[B48] McCullough M. E. Rachal K. C. Sandage S. J. Worthington E. L. Jr. Brown S. W. Hight T. L. (1998). Interpersonal forgiving in close relationships: II. Theoretical elaboration and measurement. *J. Pers. Soc. Psychol.* 75 1586–1603. 10.1037/0022-3514.75.6.1586 9914668

[B49] Moons W. G. Eisenberger N. I. Taylor S. E. (2010). Anger and fear responses to stress have different biological profiles. *Brain Behav. Immun.* 24 215–219. 10.1016/j.bbi.2009.08.009 19732822

[B50] Muller D. Judd C. M. Yzerbyt V. Y. (2005). When moderation is mediated and mediation is moderated. *J. Pers. Soc. Psychol.* 89 852–863. 10.1037/0022-3514.89.6.852 16393020

[B51] Nabi R. L. (2003). Exploring the framing effects of emotion do discrete emotions differentially influence information accessibility, information seeking, and policy preference? *Commun. Res.* 30 224–247. 10.1177/0093650202250881

[B52] Santelli A. G. Struthers C. W. Eaton J. (2009). Fit to forgive: exploring the interaction between regulatory focus, repentance, and forgiveness. *J. Pers. Soc. Psychol.* 96 381–394. 10.1037/a0012882 19159138

[B53] Skitka L. J. Bauman C. W. Aramovich N. P. Morgan G. S. (2006). Confrontational and preventative policy responses to terrorism: anger wants a fight and fear wants “them” to go away. *Basic Appl. Soc. Psychol.* 28 375–384. 10.1207/s15324834basp2804_11

[B54] Spencer S. J. Zanna M. P. Fong G. T. (2005). Establishing a causal chain: why experiments are often more effective than mediational analyses in examining psychological processes. *J. Pers. Soc. Psychol.* 89 845–851. 10.1037/0022-3514.89.6.845 16393019

[B55] Strauman T. J. Higgins E. T. (1987). Automatic activation of self-discrepancies and emotional syndromes: when cognitive structures influence affect. *J. Pers. Soc. Psychol.* 53 1004–1014. 10.1037//0022-3514.53.6.1004 3694448

[B56] Stuewig J. Tangney J. P. Heigel C. Harty L. McCloskey L. (2010). Shaming, blaming, and maiming: functional links among the moral emotions, externalization of blame, and aggression. *J. Res. Pers.* 44 91–102. 10.1016/j.jrp.2009.12.005 20369025 PMC2848360

[B57] Tangney J. P. Stuewig J. Martinez A. G. (2014). Two faces of shame the roles of shame and guilt in predicting recidivism. *Psychol. Sci.* 25 799–805. 10.1177/0956797613508790 24395738 PMC4105017

[B58] Tangney J. P. Stuewig J. Mashek D. J. (2007). Moral emotions and moral behavior. *Annu. Rev. Psychol.* 58 345–372. 10.1146/annurev.psych.56.091103.070145 16953797 PMC3083636

[B59] ten Brinke L. Adams G. S. (2015). Saving face? When emotion displays during public apologies mitigate damage to organizational performance. *Organ. Behav. Hum. Decis. Process.* 130 1–12. 10.1016/j.obhdp.2015.05.003

[B60] Tiedens L. Z. Linton S. (2001). Judgment under emotional certainty and uncertainty: the effects of specific emotions on information processing. *J. Pers. Soc. Psychol.* 81 973–988. 10.1037/0022-3514.81.6.973 11761319

[B61] Tracy J. L. Robins R. W. (2004). Putting the self into self-conscious emotions: a theoretical model. *Psychol. Inq.* 15 103–125. 10.1207/s15327965pli1502_01

[B62] Treeby M. Bruno R. (2012). Shame and guilt-proneness: divergent implications for problematic alcohol use and drinking to cope with anxiety and depression symptomatology. *Pers. Individ. Differ.* 53 613–617. 10.1016/j.paid.2012.05.011

[B63] Tsarenko Y. Tojib D. (2012). The role of personality characteristics and service failure severity in consumer forgiveness and service outcomes. *J. Mark. Manag.* 28 1217–1239. 10.1080/0267257X.2011.619150

[B64] Tsarenko Y. Tojib D. (2015). Consumers’ forgiveness after brand transgression: the effect of the firm’s corporate social responsibility and response. *J. Mark. Manag.* 31 1851–1877. 10.1080/0267257X.2015.1069373

[B65] van der Meer T. G. Verhoeven J. W. (2014). Emotional crisis communication. *Public Relat. Rev.* 40 526–536. 10.1016/j.pubrev.2014.03.004

[B66] Wolf S. T. Cohen T. R. Panter A. T. Insko C. A. (2010). Shame proneness and guilt proneness: toward the further understanding of reactions to public and private transgressions. *Self Identity* 9 337–362. 10.1080/15298860903106843

[B67] Yin D. Bond S. Zhang H. (2014). Anxious or angry? Effects of discrete emotions on the perceived helpfulness of online reviews. *MIS Q.* 38 539–560.

[B68] Yu T. Sengul M. Lester R. H. (2008). Misery loves company: the spread of negative impacts resulting from an organizational crisis. *Acad. Manag. Rev.* 33 452–472. 10.5465/AMR.2008.31193499

[B69] Zemack-Rugar Y. Bettman J. R. Fitzsimons G. J. (2007). The effects of nonconsciously priming emotion concepts on behavior. *J. Pers. Soc. Psychol.* 93 927–939. 10.1037/0022-3514.93.6.927 18072846

