# Supplementary material for: Forgiveness from Emotion Fit: Emotional Frame, Consumer Emotion, and Feeling-Right in Consumer Decision to Forgive
Source: Front Psychol. 2016 Nov 15;7:1775. doi: 10.3389/fpsyg.2016.01775 (PMC5109223; doi:10.3389/fpsyg.2016.01775)
Supplement: Supplementary file 1 [file Table_1.docx]

**APPENDIX A**

The materials of company’s crisis communication used in Study 1.

A.1 Guilt-framing crisis communication


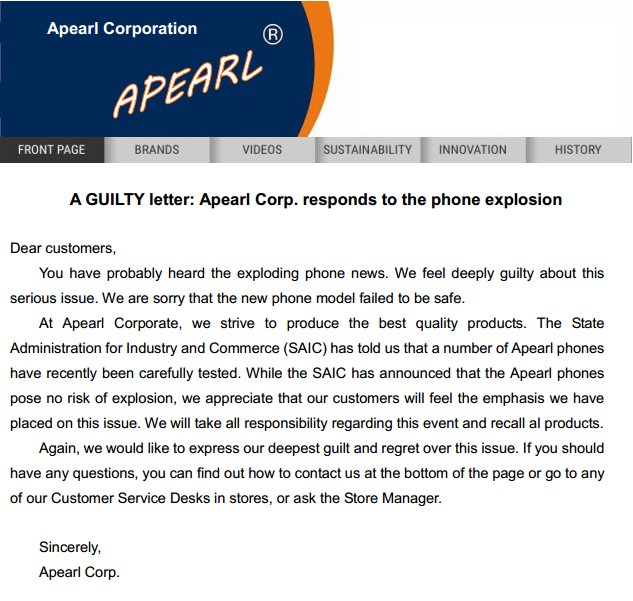


A.2 Shame-framing crisis communication


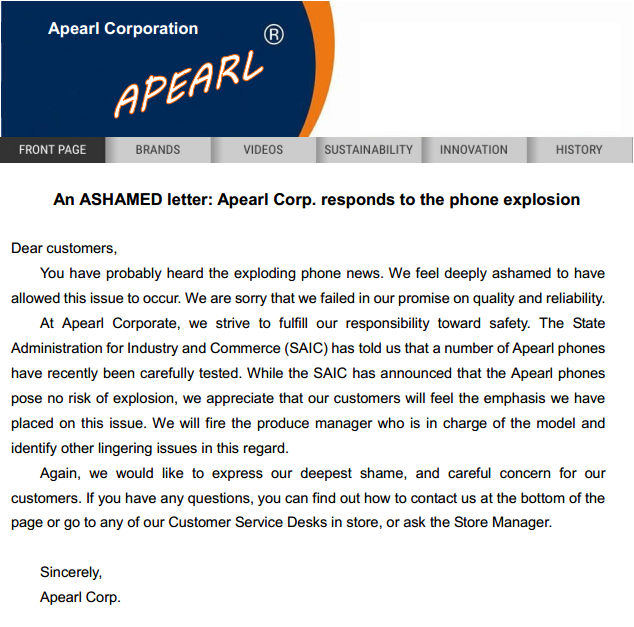


A.3 Non-emotion-framing crisis communication


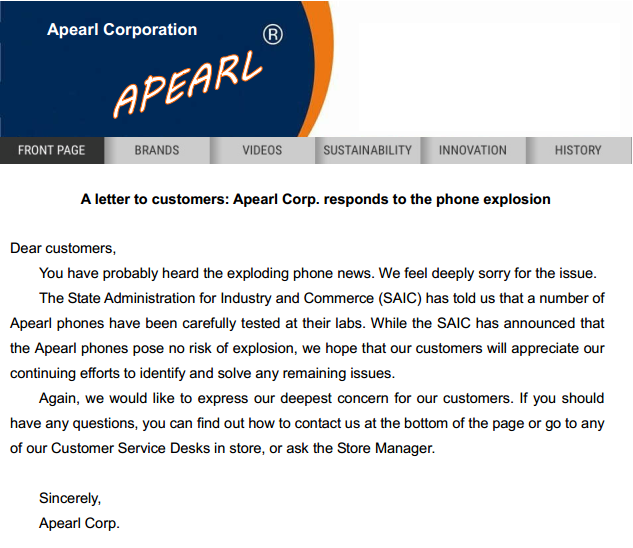


**APPENDIX B**

**TABLE A | Measures**

| **Variables** | **Factor loading** |
| --- | --- |
| **Shame (*r* = .79, *p*< .001; CR = .84; AVE = .79)** |  |
| According to the letter, the company is ashamed. | .86 |
| According to the letter, the company is humiliated. | .92 |
| **Guilt (*α* = .90; CR = .90; AVE = .75)** |  |
| According to the letter, the company is guilt. | .89 |
| According to the letter, the company is culpable. | .89 |
| According to the letter, the company is remorseful. | .81 |
| **Anger (*α* = .94; CR = .94; AVE = .83)** |  |
| I feel angry. | .90 |
| I feel irritated. | .92 |
| I feel aggravated. | .91 |
| **Fear (*r* = .66, *p*< .001; CR = .82; AVE = .70)** |  |
| I feel fearful. | .96 |
| I feel scared. | .69 |
| **TRIM-Revenge (*α* = .92; CR = .92; AVE = .70)** |  |
| I would be against to the Apearl Corporate. | .85 |
| I wish that decreased market shares would happen to the Apearl Corporate. | .88 |
| I want the Apearl Corporate to get what it deserves. | .73 |
| I want Apearl Corporate to pay. | .85 |
| I want to see Apearl Corporate losing its consumers. | .86 |
| **TRIM-Avoidance (*α* = .89; CR = .90; AVE = .58)** |  |
| I keep as much distance between me and products of Apearl Corporate. | .79 |
| I would not buy any products of Apearl Corporate. | .90 |
| I don’t trust the Apearl Corporate. | .56 |
| I avoid the Apearl Corporate. | .56 |
| I find it difficult to join in any activities launched by the Apearl Corporate. | .94 |
| I would stop consuming Apearl Corporate’s products. | .44 |
| I would not recommend the products of Apearl Corporate to others. | .96 |
| **Promotion focus (*α* = .82; CR = .82; AVE = .54)** |  |
| In my point, the current major goal of Lifewater Corporate should be to take actions to solve the problem. | .79 |
| In my point, Lifewater Corporate should focus on achieving positive outcomes in responding to the crisis. | .73 |
| At present, Lifewater Corporate should strive to fulfill the corporate vision and values. | .58 |
| In general, the major goal of Lifewater Corporate right now is to achieve the company’s ambitions. | .81 |
| **Prevention focus (*α* = .80; CR = .81; AVE = .53)** |  |
| In my point, the current major goal of Lifewater Corporate should be to avoid more occurrences of negative issues. | .79 |
| In my point, Lifewater Corporate should focus on preventing negative eventsinresponding to the crisis. | .75 |
| At present, Lifewater Corporate should strive to fulfill the corporate duties and responsibilities. | .47 |
| In general, the major goal of Lifewater Corporate right now is to avoid becoming a business failure. | .85 |
| **Feeling-right (*r* = .45, *p*< .01; CR = .61; AVE = .44)** |  |
| To what extent do you feel that the Lifewater’s letter is right? | .69 |
| To what extent do you feel that the Lifewater’s letter is correct? | .64 |
| **Forgiveness (*α* = .83; CR = .84; AVE = .57)** |  |
| I would forgive the Lifewater Corporation. | .71 |
| I would trust the Lifewater Corporation. | .87 |
| I would sympathize with the Lifewater Corporation. | .64 |
| I would excuse the Lifewater Corporation. | .77 |

Notes: *α* = Cronbach’s alpha; CR = composite reliability. All factor loadings are significant at *p*< .01. TRIM-Revenge and TRIM-Avoidance are from Study 1, other variables are from Study 2.
